# Practice variations in the management of childhood nephrotic syndrome in the Netherlands

**DOI:** 10.1007/s00431-021-03958-8

**Published:** 2021-02-03

**Authors:** Anne M. Schijvens, Lucie van der Weerd, Joanna A. E. van Wijk, Antonia H. M. Bouts, Mandy G. Keijzer-Veen, Eiske M. Dorresteijn, Michiel F. Schreuder

**Affiliations:** 1 Department of Pediatric Nephrology, Radboud University Medical Center, Radboud Institute for Molecular Life Sciences, Amalia Children’s Hospital, 804, P.O. Box 9101, 6500 HB Nijmegen, The Netherlands; 2 Department of Pediatric Nephrology, Amsterdam University Medical Center, Vrije Universiteit Amsterdam, Amsterdam, The Netherlands; 3 Department of Pediatric Nephrology, Amsterdam University Medical Center, Emma Children’s Hospital, Amsterdam, The Netherlands; 4 Department of Pediatric Nephrology, University Medical Center Utrecht, Wilhelmina Children’s Hospital, Utrecht, The Netherlands; 5 Department of Pediatric Nephrology, Erasmus MC - Sophia Children’s Hospital, Rotterdam, The Netherlands

**Keywords:** Nephrotic syndrome, Clinical practice, Pediatric nephrology, Guidelines

## Abstract

Nephrotic syndrome in childhood is a common entity in the field of pediatric nephrology. The optimal treatment of children with nephrotic syndrome is often debated. Previously conducted studies have shown significant variability in nephrotic syndrome management, especially in the choice of steroid-sparing drugs. In the Netherlands, a practice guideline on the management of childhood nephrotic syndrome has been available since 2010. The aim of this study was to identify practice variations and opportunities to improve clinical practice of childhood nephrotic syndrome in the Netherlands. A digital structured survey among Dutch pediatricians and pediatric nephrologists was performed, including questions regarding the initial treatment, relapse treatment, kidney biopsy, additional immunosuppressive treatment, and supportive care. Among the 51 responses, uniformity was seen in the management of a first presentation and first relapse. Wide variation was found in the tapering of steroids after alternate day dosing. Most pediatricians and pediatric nephrologists (83%) would perform a kidney biopsy in case of steroid-resistant nephrotic syndrome, whereas for frequent relapsing and steroid-dependent nephrotic syndrome this was 22% and 41%, respectively. Variation was reported in the steroid-sparing treatment. Finally, significant differences were present in the supportive treatment of nephrotic syndrome.

*Conclusion*: Substantial variation was present in the management of nephrotic syndrome in the Netherlands. Differences were identified in steroid tapering, use of steroid coverage during stress, choice of steroid-sparing agents, and biopsy practice. To promote guideline adherence and reduce practice variation, factors driving this variation should be assessed and resolved.

**Table:** 

**What is Known:** • *National and international guidelines are available to guide the management of childhood nephrotic syndrome.* • *Several aspects of the management of childhood nephrotic syndrome, including the choice of steroid-sparing drugs and biopsy practice, are controversial and often debated among physicians.* **What is New:** • *Significant practice variation is present in the management of childhood nephrotic syndrome in the Netherlands, especially in the treatment of FRNS, SDNS, and SRNS.* • *The recommendation on the steroid treatment of a first episode of nephrotic syndrome in the KDIGO guideline leaves room for interpretation and is likely the cause of substantial differences in steroid-tapering practices among Dutch pediatricians and pediatric nephrologists.*

**Supplementary Information:**

The online version contains supplementary material available at 10.1007/s00431-021-03958-8.

## Introduction

Nephrotic syndrome, characterized by the triad of proteinuria, hypoalbuminemia, and edema, is a common entity in the field of pediatric nephrology [1–3]. For over 60 years, steroids have been the cornerstone of the treatment of nephrotic syndrome. Other aspects of the management of childhood nephrotic syndrome are more controversial and often debated among physicians [4–7]. As consensus and evidence about the most appropriate second-line drug are lacking, the choice of therapy mostly depends on possible side effects, the physician’s personal experience with and preference for the drug, and patient circumstances. Another matter of debate is the indication to perform a kidney biopsy. In the past, many children with nephrotic syndrome underwent a kidney biopsy [8]. However, as 80–90% of the children respond to steroid therapy that could not be predicted on the biopsy results, this evolved to performing biopsies only in a selection of children with nephrotic syndrome [9, 10]. Currently, consensus has been reached on SRNS as an indication for kidney biopsy [4–6]. Moreover, kidney biopsies are generally considered in patients with atypical clinical characteristics at presentation, including onset at less than 12 months or over 12 years of age, macroscopic hematuria, low complement levels, kidney failure not related to hypovolemia, and persistent hypertension [11].

In the Netherlands, pediatric patients with steroid-sensitive nephrotic syndrome are treated by both pediatricians and pediatric nephrologists. In case of steroid resistance, patients are generally referred to a pediatric nephrologist. In the Netherlands, a national practice guideline has been available since 2010, including information on the steroid treatment of childhood nephrotic syndrome. Recommendations on kidney biopsy practice are less clear. Moreover, minimal guidance is provided on the choice of second-line immunosuppressive treatment [12]. The aim of this project is to identify practice patterns of Dutch pediatricians and pediatric nephrologists in the management of childhood nephrotic syndrome and subsequently compare the results with recommendations in the current Dutch practice guideline and international 2012 Kidney Disease: Improving Global Outcomes (KDIGO) Clinical Practice Guideline for Glomerulonephritis [12, 13].

## Methods

### Design and setting

A detailed, structured web-based survey to evaluate practice patterns in childhood nephrotic syndrome was designed. The survey questions (*n* = 72) were reviewed in detail by two pediatric nephrologists (ED and MS). The final survey was created using a web-based survey program (Castor Electronic Data Capture) and distributed by email among members of the Pediatric Nephrology section of the Pediatric Association of the Netherlands. Invitations, which were sent to 103 pediatricians and pediatric nephrologists, were repeated twice over a period of 5 months (October 2017–February 2018) to optimize response rates.

### Contents of the survey

The survey was divided into topics:

1. Management of steroid-sensitive nephrotic syndrome, including the first episode of nephrotic syndrome, infrequent relapses of nephrotic syndrome, FRNS, and SDNS.
2. Management of SRNS
3. Kidney biopsy practice
4. Supportive treatment

### Dutch pediatric nephrology practice guideline

The current Dutch practice guideline states that the first presentation of childhood nephrotic syndrome should be treated with oral prednisolone, 60 mg/m^2^ once daily for 6 weeks, with a maximum dosage of 80 mg/day, followed by 6 weeks of prednisolone 40 mg/m^2^ on alternate days [12]. In case of a relapse, daily prednisolone 60 mg/m^2^ (maximum 80 mg) is prescribed until proteinuria has resolved for 3 days, followed by 6 weeks of prednisolone 40 mg/m^2^ on alternate days, based on the original scheme from the Arbeitsgemeinschaft für Pädiatrische Nephrologie (APN) [14, 15]. For FRNS, SDNS, or SRNS, different options for steroid-sparing drugs are mentioned in the Dutch practice guideline including cyclophosphamide, cyclosporine, MMF, and levamisole.

### Definitions

In the questionnaire, definitions were based on the 2012 KDIGO Clinical Practice Guideline [13]. Infrequent relapse was defined as a relapse within 6 months of initial response, or one to three relapses in any 12-month period. The definition of FRNS includes two or more relapses within 6 months of initial response, or four or more relapses in any 12-month period. Steroid dependency was defined as two consecutive relapses during corticosteroid therapy, or within 14 days of ceasing therapy.

### Data analysis

Results are reported using the proportion of total respondents of the individual question. For the data analysis, pediatricians and pediatricians with nephrology expertise are merged into one group (pediatricians). Pediatric nephrologists in training and pediatric nephrologists are analyzed as pediatric nephrologists. Few categorical values were analyzed using a chi-squared test. GraphPad was used to create the artwork.

## Results

### Participant characteristics

In total, 103 physicians (pediatric nephrologists (*n* = 23) and pediatricians (*n* = 80)) received the questionnaire of whom 51 (50%) completed the survey. Pediatric nephrologists showed a significantly higher response rate in comparison with pediatricians (91% and 38%, respectively). Supplemental Table 1 shows the demographics of the respondents. Physicians are distributed across all regions of the Netherlands.

### Management of steroid-sensitive nephrotic syndrome

#### First episode of nephrotic syndrome

All pediatricians and pediatric nephrologists start with prednisolone treatment for a first presentation of nephrotic syndrome (Table 1). Nearly all respondents (98%) use mg/m^2^ dosing with a daily dose of 60 mg/m^2^, followed by alternate day dosing of 40 mg/m^2^. Some variation was present in the maximum dose used which did not depend on the profession, age, percentage of clinical work, or institution (Table 1, Supplemental Table 2). The only respondent who uses the mg/kg dosing prescribed 2 mg/kg for both the daily and the alternate day dosing with a maximum of 80 mg. The frequency of daily dosing ranged from once daily (68%) to 2–3 divided doses daily (26%). The majority (80%) of the pediatric nephrologists prescribes prednisolone in one daily dose, whereas for the pediatricians this was only 60% (Supplemental Table 2). Wide variation was found in the tapering of steroids after alternate day dosing for the treatment of the first episode of nephrotic syndrome. Approximately one third of the respondents, predominantly pediatricians, follow a schedule for tapering of steroids. The tapering schedules vary significantly and include a duration between 2 and 26 weeks.

**Table 1:** Steroid regimens for the first presentation and infrequent relapse of nephrotic syndrome

		First presentation	Infrequent relapse
Duration of daily steroids	6 weeks	42 (91%)	3 (7%)
	4 weeks	2 (4%)	1 (2%)
	Based on absence of proteinuria	2 (4%)	40 (91%)
Duration of alternate day steroids	6 weeks	42 (93%)	24 (56%)
	4 weeks	3 (7%)	18 (42%)
	Other	0 (0%)	1 (2%)
Maximum dose of daily steroids	100 mg	1 (2%)	
	80 mg	33 (80%)	
	60 mg	7 (17%)	
Maximum dose of alternate day steroids	100 mg	1 (2%)	
	80 mg	18 (44%)	
	60 mg	15 (37%)	
	55 mg	1 (2%)	
	50 mg	2 (5%)	
	40 mg	4 (10%)	

#### Infrequent relapse of nephrotic syndrome

For a relapse of nephrotic syndrome, the majority of the respondents prescribes prednisolone daily (60 mg/m^2^) until proteinuria is absent for 3 days, followed by 6 weeks of alternate day prednisolone therapy (40 mg/m^2^) (Table 1, Supplemental Table 2). A quarter of the respondents use a tapering schedule after alternate day dosing.

#### FRNS

**Fig. 1:**
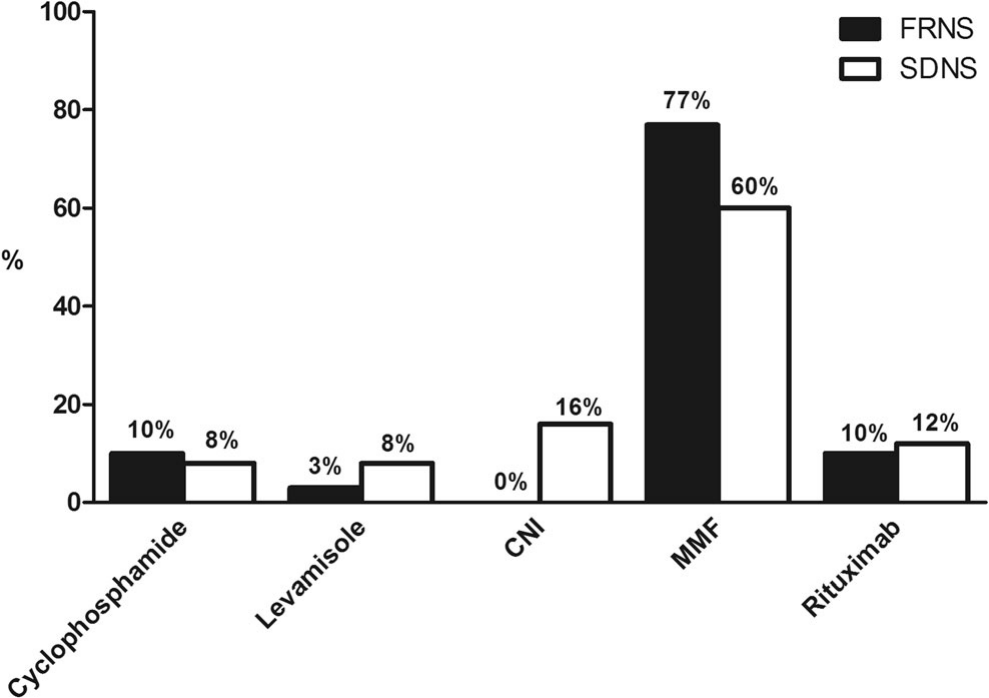
First choice of steroid-sparing drugs for FRNS and SDNS. CNI calcineurin inhibitor, MMF mycophenolate mofetil

Additional steroid-sparing drugs are prescribed by the majority of the respondents (93%) in case of FRNS. Most pediatricians indicated to consult a pediatric nephrologist in case the patient progresses to FRNS. In Fig. 1, the first choice of steroid-sparing drugs for FRNS is depicted. Table 2 shows the duration of maintenance therapy for FRNS, SDNS, and SRNS after sustained remission. Over 60% of the respondents consider tapering of steroids after the alternate day dosing regimen in FRNS.

**Table 2:** Duration of maintenance therapy for FRNS, SDNS, and SRNS after sustained remission

Duration of maintenance therapy	FRNS	SDNS	SRNS
6 months	4 (14%)	2 (10%)	1 (5%)
12 months	11 (38%)	9 (42%)	11 (52%)
24 months	11 (38%)	8 (38%)	8 (38%)
Between 12–24 months	1 (3%)	1 (5%)	–
Dependent on drug of choice	2 (7%)	1 (5%)	1 (5%)

*FRNS* frequent relapsing nephrotic syndrome, *SDNS* steroid-dependent nephrotic syndrome, *SRNS* steroid-resistant nephrotic syndrome

#### SDNS

Nearly all pediatricians indicated to consult a pediatric nephrologist in case of SDNS. Two thirds of the respondents who treat SDNS patients indicated to start with steroid-sparing drugs, whereas one third indicated to start with low-dose prednisolone maintenance therapy. Figure 1 shows the preference of steroid-sparing drugs used in SDNS.

#### Rituximab

In the Netherlands, rituximab is available for the treatment of nephrotic syndrome. Steroid toxicity was considered an indication for treatment with rituximab in 53% of the respondents. Over 40% of pediatricians and pediatric nephrologists considered FRNS and SDNS to be an indication for treatment with rituximab, however, usually not as a first choice treatment (Fig. 1). The majority of the respondents (55%) prescribe 2 dosages of 375 mg/m^2^ with an interval of 2 weeks, whereas 22% prescribes a single infusion of 375 mg/m^2^. Moreover, 38% of the respondents consider repeating the dosage(s) after B cell recovery.

### Management of SRNS

**Fig. 2:**
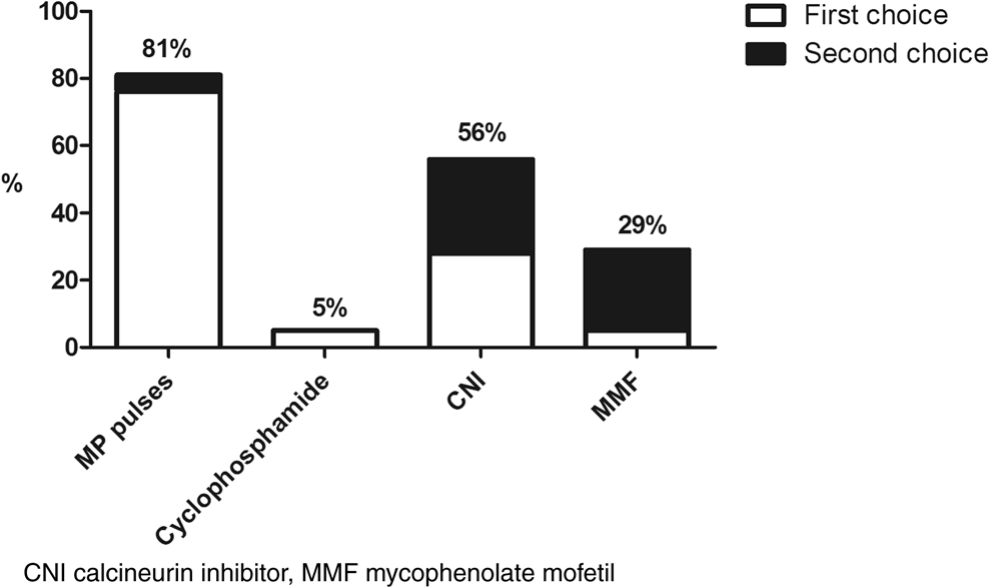
Choice of steroid-sparing drugs for SRNS.

The duration of steroid therapy before labeling a patient as steroid resistant varied widely among the respondents, ranging between 4 and 8 weeks of steroid therapy. As shown in Fig. 2, the preferred treatment for SRNS was methylprednisolone followed by a calcineurin inhibitor, with a small preference for cyclosporine over tacrolimus (57% versus 43%, respectively). Similar to FRNS and SDNS, most pediatricians indicated to consult the pediatric nephrologist in case of SRNS. Genetic analysis is performed as standard of care by 88% of the respondents. Most of the respondents taper and withdraw immunosuppressive medication in case of a genetic form of SRNS. Inhibition of the renin-angiotensin-aldosterone system (RAAS) is always initiated by 39% of respondents, whereas 26% of the respondent indicated to never or rarely prescribe RAAS inhibition in case of SRNS. KDIGO guideline adherence for the initiation of RAAS inhibition in SRNS is depicted in Supplemental Table 2. In case RAAS inhibition is initiated, 69% of the respondents prescribe monotherapy with an angiotensin-converting-enzyme (ACE) inhibitor, whereas 13% prescribes a combination of ACE inhibitors and angiotensin-II receptor blockers (ARB).

### Kidney biopsy practice

Biopsy practice varied significantly among the respondents. For SRNS, most pediatricians and pediatric nephrologists (83%) indicated to perform a kidney biopsy, whereas for FRNS and SDNS this was 22% and 41%, respectively. Only pediatricians considered performing a kidney biopsy in case of FRNS. If CNIs are used, over 50% of the respondents perform a kidney biopsy to monitor nephrotoxicity, either prior to the start of CNI, after 1–2 years of CNI use and/or in case of rising creatinine.

### Supportive treatment

#### Albumin infusions and diuretics

Albumin infusions are considered in case of low serum albumin (reported range between 10 and 25 g/l) by 10% of the respondents, if severe intravascular depletion is present (68% of respondents), or in case of a combination of the two (38% of respondents). In general, pediatricians and pediatric nephrologists indicate to use diuretics in combination with albumin infusions (always 63%, sometimes 34%), with furosemide being the preferred diuretic drug. A minority of the respondents indicated to use hydrochlorothiazide (19%) and spironolactone (3%) as preferred diuretic. Variation is present among respondents in prescribing diuretics when edema is present. Diuretics are sometimes prescribed by 59% of the respondents, whereas 36% of respondents indicated to never/hardly ever prescribe diuretics in case of edema.

#### Salt and fluid restriction

Salt restriction is “always” advised by 41% of physicians. Sixteen percent of the physicians prescribe salt restriction in case of therapy-resistant nephrotic syndrome or when edema is present. Moreover, most physicians (65%) advise fluid restriction in the acute phase of nephrotic syndrome. Fluid restriction is advised by 21% of the respondents in case of edema, whereas hyponatremia is an indication for fluid restriction for 19% of the respondents. In contrast, 14% of the respondents would never advise fluid restriction. When fluid restriction is prescribed, 39% of respondents advices 80% of normal fluid intake, a quarter recommends 60% of normal fluid intake. Finally, restriction based on insensible loss and diuresis is indicated by one third of the physicians.

#### Thrombosis prophylaxis

Thrombosis prophylaxis is considered by almost half of the respondents in case albumin levels are below 20 g/l for over a month. Moreover, a quarter of the respondents (predominantly pediatricians) indicate to prescribe thrombosis prophylaxis in case the thrombocyte count rises above a certain value (ranging between 400 and 1200 × 10^9^/l).

#### Calcium and vitamin D management

**Fig. 3:**
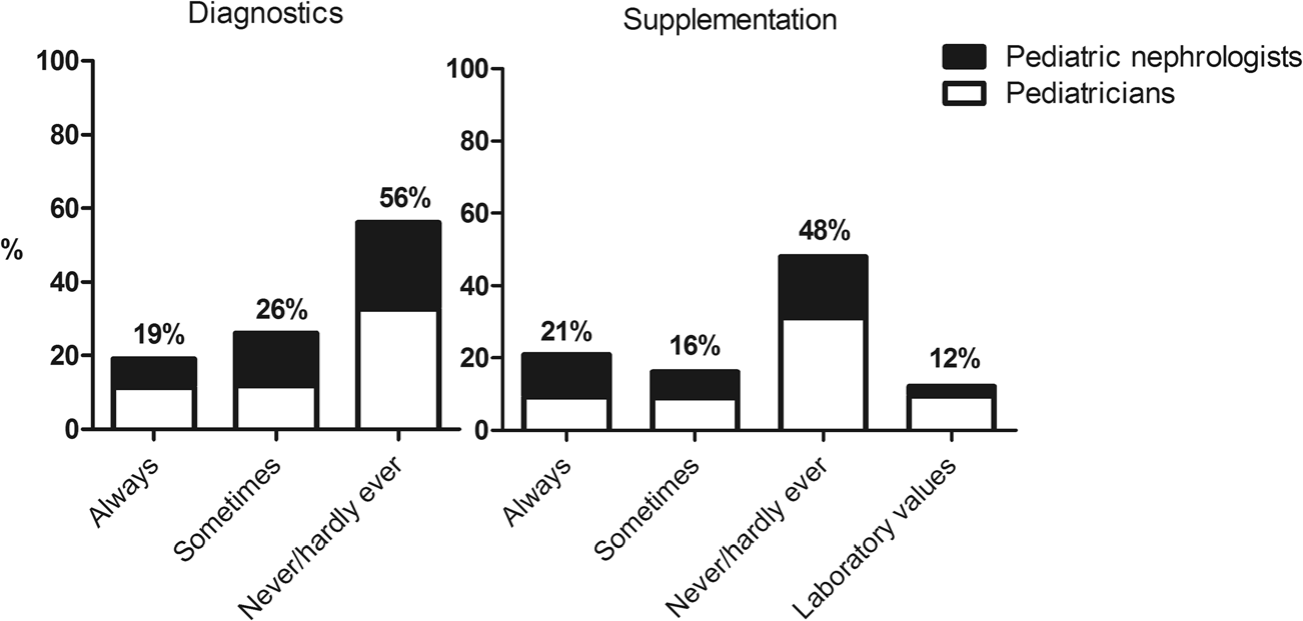
Calcium and vitamin D diagnostics and supplementation at presentation

Variation was present in the calcium and vitamin D management of nephrotic syndrome patients (Fig. 3). Most respondents do not supply calcium and/or vitamin D at the first presentation of nephrotic syndrome.

#### Stress dose steroids

After steroid treatment, stress dose steroids may be used to prevent adrenal insufficiency. A substantial portion of the participating physicians (38%) indicate to always prescribe stress dose steroids. A smaller group (31%) sometimes uses stress dose steroids whereas a quarter never does. A relatively higher percentage of pediatricians (44%) indicated to always prescribe stress dose steroids compared with pediatric nephrologists (31%). In contrast, the majority of respondents (89%) indicated to never/hardly ever use a low-dose ACTH test.

#### Varicella zoster vaccination

Finally, large variation was seen in the indication and timing of varicella zoster vaccination in patients who were not previously exposed to the virus. Indications ranged from “only in patients treated with second-line immunosuppressive agents,” “patients with low serum albumin levels,” “steroid-resistant patients,” to never and always. Over one third of the respondents indicated to vaccinate siblings of the patient in case they were not exposed to the varicella zoster virus yet, without significant practice differences in pediatricians compared with pediatric nephrologists.

## Discussion

This study shows significant practice variation in the management of childhood nephrotic syndrome in the Netherlands, especially for the treatment of FRNS, SDNS, and SRNS. Uniformity seems to be present in the management of a first presentation and infrequent relapse of nephrotic syndrome, with the exception of the tapering practices. Moreover, variation was present in the choice of steroid-sparing agents, biopsy practice, the use of a steroid coverage during stress, and supportive treatment.

As expected, minimal variation was seen in the steroid treatment of a first presentation of nephrotic syndrome in the Netherlands. The majority of the pediatricians and pediatric nephrologists follow the steroid regimen of the APN, in line with the Dutch practice guideline as well as the 2012 KDIGO Clinical Practice Guideline [12, 13]. Interestingly, variation was found in the maximum dose of steroids used. This is most likely due to different recommendations on the maximum prednisolone dose for daily steroids in the Dutch practice guideline (max. 80 mg) and 2012 KDIGO Clinical Practice Guideline (max. 60 mg) [12, 13]. Even more variation was present in the maximum dose in the alternate day dosing period, which is probably due to the lack of a maximum dose for the alternate day regimen in the Dutch practice guideline [12]. Moreover, wide variation was found in the tapering of steroids after alternate day dosing. Although tapering is not described in the Dutch practice guideline nor in the recommendations of the Gesellschaft für Pädiatrische Nephrologie, this is most likely based on the KDIGO Clinical Practice Guideline which states that daily oral prednisolone is given for 4–6 weeks followed by alternate day medication and continued for 2–5 months with tapering of the dose [12, 13, 16]. Interestingly, the recommendation of the KDIGO guideline for the treatment of a first episode of nephrotic syndrome was recently challenged by three well-conducted trials that showed no benefit of prolongation of steroid therapy beyond 3 months [17–19]. Moreover, as current steroid regimens for the treatment of nephrotic syndrome are associated with pronounced steroid associated toxicity, establishing the most effective and least toxic therapeutic regimen is important. Recently, the results of a prospective randomized pilot study were published in which the efficacy of different doses in achieving remission of nephrotic syndrome relapses was investigated in a small cohort of 30 patients with SSNS. The results suggest that a lower dose may be as safe and effective as the standard dose [20].

In case of FRNS or SDNS, steroid-sparing agents are often prescribed. Most pediatric nephrologists and pediatricians indicated that MMF was their preferred choice, with CNI as a second choice. The preferred choice of MMF is most likely based on a favorable side effect profile compared to CNI with the potential nephrotoxic effects being the most important limitation for CNI use [21, 22]. Rituximab was also considered by the Dutch pediatric nephrologists for the treatment of FRNS and SDNS. The 2012 KDIGO Clinical Practice Guideline previously stated that in children with FRNS and SDNS, there is low-quality evidence to support the use of MMF, and even very low-quality evidence for rituximab [13]. Nowadays, both MMF and rituximab are valuable agents in the treatment of SDNS [21, 23]. Variation was present among our respondents on the rituximab regimen used. Similarly, substantial variations in rituximab dose are present worldwide, ranging from 375 to 1500 mg/m^2^ per treatment course [24, 25]. Recently, Chan et al. [23] reviewed the effect of patient factors, rituximab dose, and use of maintenance immunosuppression on treatment outcomes. Debate exists on the indications for rituximab use. Nowadays, rituximab is often used in patients with FRNS or SDNS that typically tried several non-steroid immunosuppressants. Using rituximab as a first-line treatment option could be considered; however, long-term safety and efficacy results of this approach remain to be established. Strict monitoring is essential, especially in young children, to timely identify severe adverse events including persistent hypogammaglobulinemia. Finally, large prospective studies are needed to identify the exact indications and optimal rituximab regimen in patients with nephrotic syndrome [23]. After this questionnaire was sent, new evidence regarding the added value of levamisole in steroid-sensitive nephrotic syndrome became available [26], which consequently was not reflected in this survey. With continuously developing literature, both the 2010 Dutch practice guideline and 2012 KDIGO Clinical Practice Guideline need updating, and indeed a new KDIGO guideline is currently under revision.

For SRNS, variation was present in the definition used, with a wide range in the duration of prednisone treatment before a patient is labeled steroid resistant. This variation in the definition used is a well-known issue and remains a matter of debate [27]. For the treatment of SRNS, our respondents mostly chose methylprednisolone pulses in combination with CNIs. This is consistent with previous findings on preferred SRNS treatment [4–6]. In the Cochrane review on treatment of SRNS, CNIs are considered to increase the likelihood of complete or partial remission compared with placebo or cyclophosphamide [28]. The necessity of corticosteroids as an additive to CNI therapy in SRNS is unknown. The 2012 KDIGO Clinical Practice Guideline suggests a combination of low-dose corticosteroid therapy and CNI therapy with tapering of the dose to the lowest level that maintains remission is recommended [13]. Similarly, the current recommendation of the IPNA includes tapering of prednisolone once the diagnosis of SRNS is established with discontinuation of prednisolone therapy after 6 months [27]. Finally, RAAS inhibition with either ACE inhibitors or ARBs was recommended in both the 2012 KDIGO Clinical Practice Guideline and 2020 IPNA clinical practice recommendations [27]. The use of RAAS inhibition in SRNS varied among our respondents, which leaves room for improvement.

Variation was found in biopsy practice, especially in cases of FRNS and SDNS. Similar to our results, Samuel et al. [4] showed that 97% of the physicians would perform a kidney biopsy in case of SRNS and almost one fifth would perform a biopsy with FRNS. According to the 2012 KDIGO Clinical Practice Guideline, a biopsy is indicated in children who fail to respond to corticosteroids after one or more remissions, children with a high suspicion for a different underlying kidney pathology, or children receiving CNI with a declining kidney function [13]. Respondents who indicated to recommend a kidney biopsy in case of FRNS or SDNS were mostly pediatricians. Importantly, most pediatricians indicated to consult a pediatric nephrologist in case of FRNS, SDNS, or SRNS. In the Netherlands, kidney biopsies are only performed by pediatric nephrologists in tertiary care centers. As FRNS and SDNS are not considered a direct indication to perform a kidney biopsy, information for pediatricians on the indications to perform a kidney biopsy should be optimized, as this will lead to better expectation management of the patients in case of referral to a pediatric nephrologist.

Substantial variation was found in the use of tapering regimens and additional steroids in stress situations to prevent adrenal insufficiency in pediatric nephrotic syndrome patients. In the current Dutch practice guideline and 2012 KDIGO Clinical Practice Guideline, there is no mention of stress dose steroids. In line with the 2019 guideline on steroid therapy of the Pediatric Association of the Netherlands [29], no relevant literature is available to guide clinicians on this issue. Nevertheless, based on physiological mechanisms, it is advised to consider a tapering regimen when steroid treatment duration exceeds 14 days and growth impairment or cushingoid features are present or strong CYP3A4 inhibitors are used. Consequently, the recommendation is to perform a low-dose ACTH test 3 months after discontinuation of steroids using a low-dose ACTH test in patients requiring a tapering schedule [29]. Recently, Abu Bakar et al. [30] reported the results of a study on adrenal insufficiency in children with nephrotic syndrome. Sixty-two percent (23/37) of the children showed signs of steroid toxicity, whereas only 35% of children (all below 5 years of age) had test results consistent with hypothalamic-pituitary-adrenal axis suppression. These results indicate that, especially in younger children, screening for adrenal insufficiency might be useful. Varicella infection may lead to life-threatening disease in children receiving immunosuppressive drugs. Large variation was present among respondents in the indication and timing of varicella zoster vaccination in patients who were not previously exposed to the virus. The 2012 KDIGO guideline recommends offering varicella immunization to children with steroid-sensitive nephrotic syndrome who are not receiving immunosuppressive or cytotoxic agents other than low-dose daily (< 20mg) or alternate day (< 40 mg) prednisone [13]. Finally, the majority of the respondents indicated not to supply calcium and/or vitamin D at the first presentation of nephrotic syndrome. In both the 2012 KDIGO guideline and the Dutch clinical practice guideline, there is no mention of vitamin D supplementation. In 2017, Yadav et al. [31] showed a decrease in bone mineral density with steroid treatment and a beneficial role of calcium and vitamin D supplementation even during the first episode of nephrotic syndrome. Nevertheless, the optimal dose, frequency of administration, and duration remain to be elucidated [32].

A limitation of this study is the response rate of 50%, which may have introduced reporting bias. Nearly all pediatric nephrologists completed the survey, whereas for the pediatricians this was only 38%, which may be based on the low incidence of nephrotic syndrome in childhood [2]. Furthermore, our study is based on survey results, rather than actual data of clinical practice.

To conclude, significant practice variation is present in the management of childhood nephrotic syndrome in the Netherlands and differences were identified between the results of the survey and to the Dutch practice guideline and international KDIGO Clinical Practice Guideline. Most variation can be explained by the lack of consensus due to the absence of well-performed randomized controlled trials in this patient group. Therefore, effort should be made to collaborate in international randomized controlled trials to improve evidence-based management of children with nephrotic syndrome. Moreover, with the recent literature and the subsequent new international guidelines and Cochrane reviews, the Dutch guideline needs updating [12]. After such an update, it is important to promote the adherence to guidelines and thereby reduce practice variation. In the Netherlands, the Working Group idiopathic Nephrotic syndrome should play a role in the dissemination of the new guideline among pediatricians and pediatric nephrologists throughout the country.

## Supplementary Information

ESM 1 (PDF 118 kb)

## Data Availability

Data available upon request

## Abbreviations

ACE: Angiotensin-converting-enzyme
APN: Arbeitsgemeinschaft für Pädiatrische Nephrologie
ARB: Angiotensin-II receptor blocker
CNI: Calcineurin inhibitor
FRNS: Frequent relapsing nephrotic syndrome
KDIGO: Kidney disease improving global outcomes
MMF: Mycophenolate mofetil
RAAS: Renin-angiotensin-aldosterone system
SDNS: Steroid-dependent nephrotic syndrome
SRNS: Steroid-resistant nephrotic syndrome
