# Supplementary material for: Practice variations in the management of childhood nephrotic syndrome in the Netherlands
Source: Eur J Pediatr. 2021 Feb 3;180(6):1885–94. doi: 10.1007/s00431-021-03958-8 (PMC8105198; doi:10.1007/s00431-021-03958-8)
Supplement: Supplementary file 1 — (PDF 118 kb) [file 431_2021_3958_MOESM1_ESM.pdf]

Supplementary Information

Practice variations in the management of childhood nephrotic syndrome in the Netherlands

Anne M. Schijvens, MD[1], Lucie van der Weerd, BSc[1*], Joanna A.E. van Wijk, MD PhD[2], Antonia H.M. Bouts, MD PhD[3], Mandy Keijzer-Veen, MD PhD[4], Eiske M. Dorresteijn, MD[5], Michiel F. Schreuder, MD PhD[1].

[1]Radboud university medical center, Radboud Institute for Molecular Life Sciences, Amalia Children's Hospital, Department of Pediatric Nephrology, Nijmegen, The Netherlands

[2]Amsterdam UMC, Vrije Universiteit Amsterdam, Department of Pediatric Nephrology, Amsterdam, The Netherlands

[3]Amsterdam UMC, University of Amsterdam, Emma Children's Hospital, Department of Pediatric Nephrology, Amsterdam, The Netherlands

[4]University Medical Center Utrecht, Wilhelmina Children's Hospital, Department of Pediatric Nephrology, Utrecht, The Netherlands

[5]Erasmus MC - Sophia Children's Hospital, Department of Pediatric Nephrology, Rotterdam, The Netherlands

Anne.schijvens@radboudumc.nl

Journal: European Journal of Pediatrics

Table of contents

**1**	**Supplemental Table 1** Demographics of the respondents
**2**	**Supplemental Table 2** Factors associated with guideline adherence for several items of the questionnaire

Table 1 Demographics of the respondents

**Characteristic**	**Category**	**N (%)**[a]
Gender	Male	21 (41%)
	Female	30 (59%)
Age	<41 years	9 (18%)
	41-50 years	22 (44%)
	51-60 years	15 (30%)
	>60 years	4 (8%)
Profession	General pediatrician	13 (26%)
	Pediatrician with focus on pediatric nephrology	17 (33%)
	Pediatric nephrologist	20 (39%)
	Pediatric nephrologist in training	1(2%)
Experience[b]	0-5 years	7 (19%)
	5-10 years	8 (23%)
	10-15 years	7 (19%)
	>15 years	14 (39%)
Clinical practice vs other responsibilities (e.g. research)	<50% clinical work	2 (4%)
	50% clinical work	5 (10%)
	>50% clinical work	44 (86%)
Institution	Academic hospital	18 (35%)
	General hospital	33 (65%)

[a]Some respondents did not answer all questions, therefore the number (N) does not always add up to N=51
[b]Information only available for pediatric nephrologists (in training) and pediatricians with focus on pediatric nephrology

Supplemental Table 1. Factors associated with guideline adherence for several items of the questionnaire

Characteristic	Category	Dutch guideline adherence % (n)	p-value	KDIGO guideline adherence % (n)	p-value
**Tapering of steroids first presentation of nephrotic syndrome**					
Profession	Pediatrician	67% (20)	0.26	33% (10)	0.26
	Pediatric nephrologist	81% (17)		19% (4)	
Age group	>48 years	75% (18)	0.65	25% (6)	0.65
	≤48 years	69% (18)		31% (8)	
Clinical practice versus other responsibilities	>50% clinical work	73% (32)	1.00	27% (12)	1.00
	≤50% clinical work	71% (5)		29% (2)	
Institution	Academic hospital	78% (14)	0.74	22% (4)	0.74
	General hospital	70% (23)		30% (10)	
**Steroid treatment relapse of nephrotic syndrome**					
Profession	Pediatrician	58% (14)	0.21	88% (21)	1.00
	Pediatric nephrologist	39% (7)		89% (16)	
Age group	>48 years	58% (11)	0.43	84% (16)	0.65
	≤48 years	46% (10)		91% (20)	
Clinical practice versus other responsibilities	>50% clinical work	53% (19)	0.66	89% (32)	0.56
	≤50% clinical work	33% (2)		83% (5)	
Institution	Academic hospital	38% (6)	0.20	88% (14)	1.00
	General hospital	58% (15)		89% (23)	
**Frequency of daily dosing**					
Profession	Pediatrician	100% (30)	NA	60% (18)	0.14
	Pediatric nephrologist	100% (20)		80% (16)	
Age group	>48 years	100% (24)	NA	63% (15)	0.31
	≤48 years	100% (25)		76% (19)	
Clinical practice versus other responsibilities	>50% clinical work	100% (43)	NA	72% (31)	0.19
	≤50% clinical work	100% (7)		43% (3)	
Institution	Academic hospital	100% (18)	NA	83% (15)	0.08
	General hospital	100% (32)		59% (19)	
**Maximum daily dose**					
Profession	Pediatrician	83% (19)	1.00	13% (3)	1.00
	Pediatric nephrologist	83% (15)		17% (3)	
Age group	>48 years	90% (17)	0.41	5% (1)	0.19
	≤48 years	76% (16)		24% (5)	
Clinical practice versus other responsibilities	>50% clinical work	86% (30)	0.29	11% (4)	0.21
	≤50% clinical work	67% (4)		33% (2)	
Institution	Academic hospital	81% (13)	1.00	19% (3)	0.66
	General hospital	84% (21)		12% (3)	
**Maximum alternate day dose**					
Profession	Pediatrician	ND	ND	9% (2)	1.00
	Pediatric nephrologist	ND		12% (2)	
Age group	>48 years	ND	ND	6% (1)	0.61
	≤48 years	ND		14% (3)	
Clinical practice versus other responsibilities	>50% clinical work	ND	ND	9% (3)	0.49
	≤50% clinical work	ND		17% (1)	
Institution	Academic hospital	ND	ND	7% (1)	1.00
	General hospital	ND		12% (3)	
**Definition steroid resistant nephrotic syndrome**					
Profession	Pediatrician	64% (7)	0.18	9% (1)	0.62
	Pediatric nephrologist	38% (6)		25% (4)	
Age group	>48 years	54% (7)	0.70	15% (4)	1.00
	≤48 years	46% (6)		15% (2)	
Clinical practice versus other responsibilities	>50% clinical work	55% (12)	0.33	23% (5)	0.55
	≤50% clinical work	20% (1)		0% (0)	

Institution	Academic hospital	36% (5)	0.18	21% (4)	1.00
	General hospital	62% (8)		15% (2)	
**RAAS inhibition in steroid resistant nephrotic syndrome patients**					
Profession	Pediatrician	ND	ND	31% (4)	0.39
	Pediatric nephrologist	ND		47% (7)	
Age group	>48 years	ND	ND	36% (4)	1.00
	≤48 years	ND		44% (7)	
Clinical practice versus other responsibilities	>50% clinical work	ND	ND	33% (8)	0.27
	≤50% clinical work	ND		75% (3)	
Institution	Academic hospital	ND	ND	46% (6)	0.49
	General hospital	ND		33% (5)	

NA, not applicable; ND, no data (no recommendation available in the guideline); RAAS, renin-angiotensin-aldosterone system
